# Deep learning and data fusion to estimate surface soil moisture from multi-sensor satellite images

**DOI:** 10.1038/s41598-023-28939-9

**Published:** 2023-02-08

**Authors:** Abhilash Singh, Kumar Gaurav

**Affiliations:** Fluvial Geomorphology and Remote Sensing Laboratory, Department of Earth and Environmental Sciences, Indian Institute of Science Education and Research, Bhopal, 462066 Madhya Pradesh India

**Keywords:** Environmental social sciences, Hydrology

## Abstract

We propose a new architecture based on a fully connected feed-forward Artificial Neural Network (ANN) model to estimate surface soil moisture from satellite images on a large alluvial fan of the Kosi River in the Himalayan Foreland. We have extracted nine different features from Sentinel-1 (dual-polarised radar backscatter), Sentinel-2 (red and near-infrared bands), and Shuttle Radar Topographic Mission (digital elevation model) satellite products by leveraging the linear data fusion and graphical indicators. We performed a feature importance analysis by using the regression ensemble tree approach and also feature sensitivity to evaluate the impact of each feature on the response variable. For training and assessing the model performance, we conducted two field campaigns on the Kosi Fan in December 11–19, 2019 and March 01–06, 2022. We used a calibrated TDR probe to measure surface soil moisture at 224 different locations distributed throughout the fan surface. We used input features to train, validate, and test the performance of the feed-forward ANN model in a 60:10:30 ratio, respectively. We compared the performance of ANN model with ten different machine learning algorithms [i.e., Generalised Regression Neural Network (GRNN), Radial Basis Network (RBN), Exact RBN (ERBN), Gaussian Process Regression (GPR), Support Vector Regression (SVR), Random Forest (RF), Boosting Ensemble Learning (Boosting EL), Recurrent Neural Network (RNN), Binary Decision Tree (BDT), and Automated Machine Learning (AutoML)]. We observed that the ANN model accurately predicts the soil moisture and outperforms all the benchmark algorithms with correlation coefficient (R = 0.80), Root Mean Square Error (RMSE = 0.040 $\mathrm {m^3/m^3}$), and bias = 0.004 $\mathrm {m^3/m^3}$. Finally, for an unbiased and robust conclusion, we performed spatial distribution analysis by creating thirty different sets of training-validation-testing datasets. We observed that the performance remains consistent in all thirty scenarios. The outcomes of this study will foster new and existing applications of soil moisture.

## Introduction

Surface soil moisture is widely used in agriculture, forestry, hydrology, flood and drought prediction, and climate change studies^1^. Depending on the applications, its measurement is required at different spatial (i.e., local, regional, or global), and temporal scales. At the local scale, soil moisture can be measured in the field using dielectric probes/sensors^2^, such as; the Time Domain Reflectometry (TDR), Frequency Domain Reflectometry (FDR), and Neutron Probe (NP). These instruments provide highly precise measurements of the surface soil moisture content at different depths. Currently, about 71 International Soil Moisture Networks (ISMN) with more than 2800 operating stations (status as of August 2021) are available worldwide. They provide near real-time point measurements of soil moisture. However, presently the global coverage of the soil moisture network stations are non-uniformly distributed. This causes a data gap, especially in the regions where soil moisture measurement stations do not exist, and if exist, they are sparsely distributed. To overcome these issues, researchers proposed to use satellite images to estimate soil moisture at regional and global scales^2–6^. For example, the European Space Agency (ESA) launched the Soil Moisture and Ocean Salinity (SMOS) mission^7^ in November 2009. The SMOS satellite carries an interferometric radiometer that operates at L-band (1.4 GHz). It provides global surface soil moisture at three days revisit time with a spatial resolution of about 30–50 km. Later in January 2015, the National Aeronautics and Space Administration (NASA) launched the Soil Moisture Active Passive (SMAP) under Earth System Science Pathfinder (ESSP) mission^8^. This is equipped with L-band radar sensor and radiometers. It provides daily soil moisture products at a spatial resolution 1–36 km^9^. Despite their global coverage, data voids are present in the SMOS and the SMAP products, particularly at the complex topography, snow-covered, and densely vegetated regions^10,11^.

To overcome this, researchers explored the potential of Synthetic Aperture Radar (SAR) microwave remote sensing techniques to estimate soil moisture at high spatial and temporal resolutions^12–14^. In the microwave regions, surface soil exhibits a permittivity ($\mathrm {\epsilon }$) gradient between the dry and wet soil. For example, the permittivity ($\epsilon$) of dry soil is $\approx$ 2, and for water, it is $\approx$ 80^15–17^.

Several backscattering models (empirical, semi-empirical, and theoretical) have been proposed to estimate soil moisture from SAR images^18–23^. These models require quad polarised [i.e., Horizontal-Horizontal (HH), Vertical-Vertical (VV), Horizontal-Vertical (HV), and Vertical-Horizontal (VH)] microwave SAR images along with the sensor properties (i.e., wavelength and incidence angle) to estimate soil moisture. One can retrieve soil permittivity and soil roughness parameters from the inversion of the above models. The soil permittivity can be used in Topp’s model^24^ to obtain soil moisture^25–33^. The quad polarised SAR images are often not available. To overcome this limitation, the existing backscattering models have been modified according to dual polarised SAR images by eliminating one of the unknown parameter (surface roughness) using the in-situ measurements^14,34–38^. The resulting model will have one-equation with one-unknown, which can be solved to get the soil moisture. However, the accuracy of the retrieved soil moisture depends on the accuracy of the surface roughness^39^. These backscattering models assume ideal soil conditions and consider the soil attributes (i.e., soil moisture and roughness) as a stationary process^40^. Such assumptions get violated in the regions where surface topography is complex. Further, these models are validated under control environments at a finer scale. Hence, they may not concede well over a region with significant intra-field variations^41,42^.

Recently, machine and deep learning models have emerged as an efficient tool to predict surface soil moisture at high spatial and temporal scales^43–45^. Unlike physical models, machine or deep learning models are data-driven. They combine different relevant input features to map the output. For instance, brightness temperature, SAR backscatter, sensor properties, geographical information, and meteorological variables can be used as input features to setup a machine learning model^4,46,47^. During the training process, a machine learning model learns the soil moisture dynamics solely from the input data. Once the model is developed, its performance can be evaluated from the unseen data. ANN is a widely used machine learning model to estimate soil moisture^48–51^.

Ahmad et al.^4^ used Tropical Rainfall Measuring Mission (TRMM) and Advanced Very High-Resolution Radiometer (AVHRR) data to estimate soil moisture at 12 km spatial resolution on a daily scale. They trained SVR models at six different sites in their study area by using three input features (i.e., backscatter values, incidence angle from TRMM, and normalised difference vegetation index from AVHRR). For all the sites, they reported that the correlation coefficient ranges from 0.34 to 0.77 and RMSE < 2$\%$. They have also compared the SVR output with ANN and the Multivariate Linear Regression model (MLR). They concluded that the SVR model outperforms ANN and MLR. Santi et al.^50^ proposed the ANN-based approach to estimate daily soil moisture at 10 km spatial resolution. They used backscatter, local incidence angle, azimuth angle, Latitude, Longitude information from Advanced Scatterometer (ASCAT), and soil moisture information from International Soil Moisture Network (ISMN) to train the ANN model. They reported that ANN performs well on the testing data sets with R = 0.82 and RMSE = 0.04 $\mathrm {m^3/m^3}$. Lee et al.^43^ applied twenty-five different variants of an ANN-based deep learning model to estimate daily soil moisture at 4 km spatial resolution. They used NDVI, outgoing longwave radiation (OLR), solar insolation (INS), broadband albedo (AL), and integrated multi-satellite retrievals for global precipitation measurement (IMERG) as input features to train the models. They observed that the ANN with four hidden layers and 600 neurons in each layer outperforms all other variants with R = 0.89, RMSE = 3.825%, and bias = -0.039. Santi et al.^52^ used the ANN model to estimate surface soil moisture from fully polarimetric C-band RADARSAT-2. They used different features from the linear (HV, HH, and VV) and circular polarised data (Stokes vector, RH-RV phase difference, Shannon entropy polarimetric component, alpha angle, RH-RV correlation coefficient, conformity coefficient, and circular polarization ratio).

Adab et al.^53^ used a machine learning model to estimate surface soil moisture solely from the optical and thermal images of Landsat-8. They selected Blue (Band-2), Green (Band-3), Red (Band-4), NIR (Band-5), SWIR1 (Band-6), SWIR2 (Band-7), and Land Surface Temperature (LST) as the potential features to train and validate four different machine learning (RF, SVR, ANN, and elastic net) algorithms. They concluded that the RF is able to predict surface soil moisture accurately. The final soil moisture product has a spatial resolution of 30 m and a temporal resolution same as the Landsat-8 (i.e., 16 days). Datta et al.^54^ proposed a regression-based machine learning model to estimate surface soil moisture from Sentinel-1 images at 12 days temporal resolution. They used VV, and VH polarised images as the input features to train the models (RF, SVR, linear regression, multiple linear regression, and K-nearest neighbors). They used 40 and 16 in-situ samples to train and validate the models, respectively. They observed RF outperforms all the other models with R = 0.93 and RMSE = 0.03 $\mathrm {m^3/m^3}$. Greifeneder et al.^55^ applied the gradient-boosted regression tree-based approach on the Google Earth Engine platform to estimate surface soil moisture at a 50 m spatial scale at every 12 days. They used soil moisture data from ISMN and features from Landsat-8 and Sentinel-1 to train the machine learning model. They reported that the model performs reasonably well on the test data with R = 0.90 and RMSE = 0.04 $\mathrm {m^3/m^3}$.

The studies discussed above suggest a trade-off between the spatial and temporal resolutions of the soil moisture products. High temporal resolution soil moisture products are available at coarser spatial resolution and vice-versa. This depends on the selection of input features and soil moisture products used to train the machine learning model. Training machine learning models with the in-situ measured soil moisture by utilising the potential of multi-sensor Sentinel-1 (A & B) concurrently with multi-sensor Sentinel-2 (A & B) can provide soil moisture product at optimal spatial and temporal resolutions. This study aims to improve the spatial (60 m) and temporal (6 days) resolutions of the soil moisture product through dual polarised Sentinel-1 backscatter, red and near-infrared reflectance from Sentinel-2, and SRTM elevation data by using data fusion and a deep learning approach. We propose a novel architecture based on a fully connected feed-forward ANN to estimate surface soil moisture at high spatial and temporal resolutions from microwave (Sentinel-1), optical (Sentinel-2), and topographic (SRTM-DEM) data. For training the ANN architecture, initially, we have selected seven satellite-derived features as input predictors. We have also generated two new synthetic features through linear data fusion of existing features. We used the in-situ measured surface soil moisture as a response variable. We trained the model by using the input features as predictors and in-situ measured surface soil moisture as response. Finally, we compared the performance of our model with the benchmark algorithms (GRNN, RBN, ERBN, GPR, SVR, RF, Boosting EL, RNN, BDT, and AutoML) and selected the best model to estimate surface soil moisture on the Kosi Fan.

## Study area

**Figure 1:**
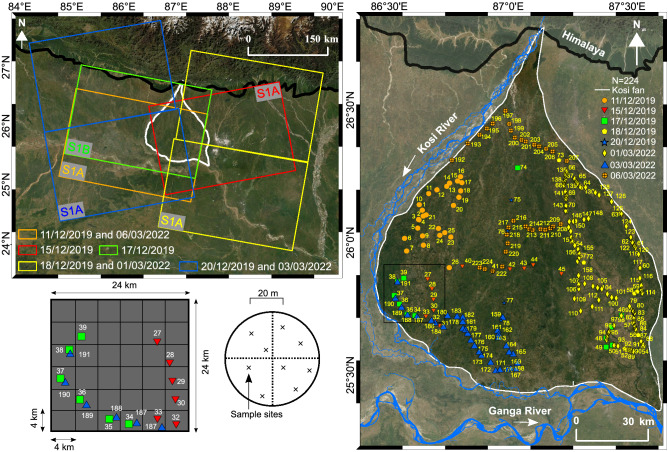
Image on the top left panel shows the rectangular footprints of the Sentinel-1 satellite images of different dates. The boundaries of the footprints are available at the European Space Agency Copernicus Open Access Hub website (https://scihub.copernicus.eu/). Image on the right panel shows the in-situ measured locations with sample ID (in different shapes) and their acquisition dates. The square grid on the bottom left panel illustrates the random sampling strategy. The maps are created in ArcGIS v10.8 software available at Environmental Systems Research Institute (ESRI) website (https://www.esri.com/en-us/arcgis/about-arcgis/overview).

**Figure 2:**
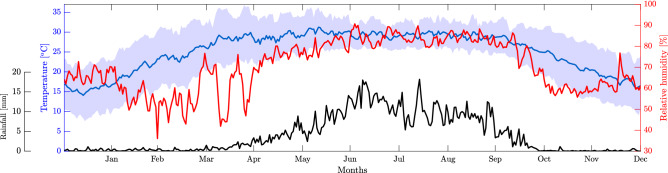
Time series plot (daily average) of the rainfall, temperature, and relative humidity over the study area from 1980 to 2018. The limits of shades in blue represent the maximum and minimum (temperature) values.

We conducted this study on the Kosi Fan in the Himalayan Foreland in north Bihar plain, India (Fig. 1). It is one of the largest fluvial fan, spread over an area of about $\mathrm {10,351 \; km^2}$. This is a result of frequent avulsions of the Kosi River channels. In the last three centuries, the Kosi River has migrated about 150 km^56–59^. During the process of migration, the river has deposited its sediments and built a large fan like structure. This fan has been active since the Holocene^60^. The Kosi Fan surface is composed of homogeneous quartz grains with a median size varying from medium sand (300 $\mathrm { \upmu m}$) to fine sand (100 $\mathrm { \upmu m}$) near its apex and toe, respectively^59,61^. The topography of the Kosi Fan is nearly flat with a small gradient from $\mathrm {8 }\times 10^{-4}$ in the proximal to $\mathrm {6 }\times 10^{-5}$ in the distal part^59^. The Kosi Fan falls in a tropical climatic zone. The minimum and maximum average annual temperature vary in a range between 18 to 32 $^\circ$C. The temperature is maximum during the summer and approaches minimum during the winter (Fig. 2). The relative humidity ranges between 30 and 90%. It is maximum (70–90%) during the monsoon (i.e., June–September) and minimum (30%) in the early summer (i.e., March–April). On an average, the Kosi Fan receives about 1484 mm rainfall annually^62,63^. Most of the rainfall (about 80%) occurs during the Indian Summer Monsoon (June–September). The groundwater on the Kosi Fan remains shallow throughout. It varies from 1.8 to 8.1 m and 1.0 to 6.4 m below ground level (bgl) during the pre-and post-monsoon periods, respectively (http://cgwb.gov.in/). Apart from this, one can see numerous waterlogged patches and isolated channels throughout the Kosi Fan. Most part of the Kosi Fan gets flooded every year during the monsoon period. The dominant landuse and landcover types are agricultural ($\approx$ 84%), surface water bodies ($\approx$ 9%), and built-up areas ($\approx$ 7%)^64^.

Altogether, the Kosi Fan is an ideal field site to study soil moisture variability. Such knowledge can be very useful for better planning of agriculture, flood, drainage congestion, and waterlogging predictions on the Kosi Fan.

## Datasets

**Table 1:** Detailed descriptions of the Sentinel-1/2 images.

Sentinel-1				
Date (dd/mm/yyyy)	Polarisation	Incidence angle ($^o$)	Pixel size (m $\times$ m)	Pass
11/12/2019	Dual (VH, VV)	38.6	10 $\times$ 10	Descending
15/12/2019	Dual (VH, VV)	38.5	10 $\times$ 10	Ascending
17/12/2019	Dual (VH, VV)	38.4	10 $\times$ 10	Descending
18/12/2019	Dual (VH, VV)	38.5	10 $\times$ 10	Descending
20/12/2019	Dual (VH, VV)	38.5	10 $\times$ 10	Ascending
01/03/2022	Dual (VH, VV)	38.4	10 $\times$ 10	Descending
03/03/2022	Dual (VH, VV)	38.5	10 $\times$ 10	Ascending
06/03/2022	Dual (VH, VV)	38.6	10 $\times$ 10	Descending

Sentinel-2				
Date (dd/mm/yyyy)	Orbit number and direction	Bands	Wavelength (nm)	Spatial Resolution (m)
09/12/2019	76, Descending	4, 8	646–685, 774–907	10
03/03/2022	76, Descending	4, 8	646–685, 774–907	10

### Satellite

We use publicly available Sentinel-1 (SAR), Sentinel-2 (optical) images, and digital elevation model (DEM) from the shuttle radar topographic mission (SRTM). We downloaded the Sentinel images from official website of the European Space Agency (https://scihub.copernicus.eu/) and SRTM-DEM from the Geological Survey (USGS) website (https://earthexplorer.usgs.gov). Table 1 reports the detailed descriptions of the Sentinel images.

European Space Agency (ESA) launched the Sentinel-1A (on 3rd April 2014) and Sentinel-1B missions (on 25th April 2016) as two-satellite constellation under the Copernicus Programme (formerly known as Global Monitoring for Environment and Security). The revisit time of these satellites is 12 days. However, when considered together, a revisit time of six days can be achieved^14,65^. Sentinel-1A and 1B operates at a frequency of 5.405 GHz. At this frequency, microwave signals can penetrate up to 5 cm below the dry soil column^66–68^.

**Figure 3:**
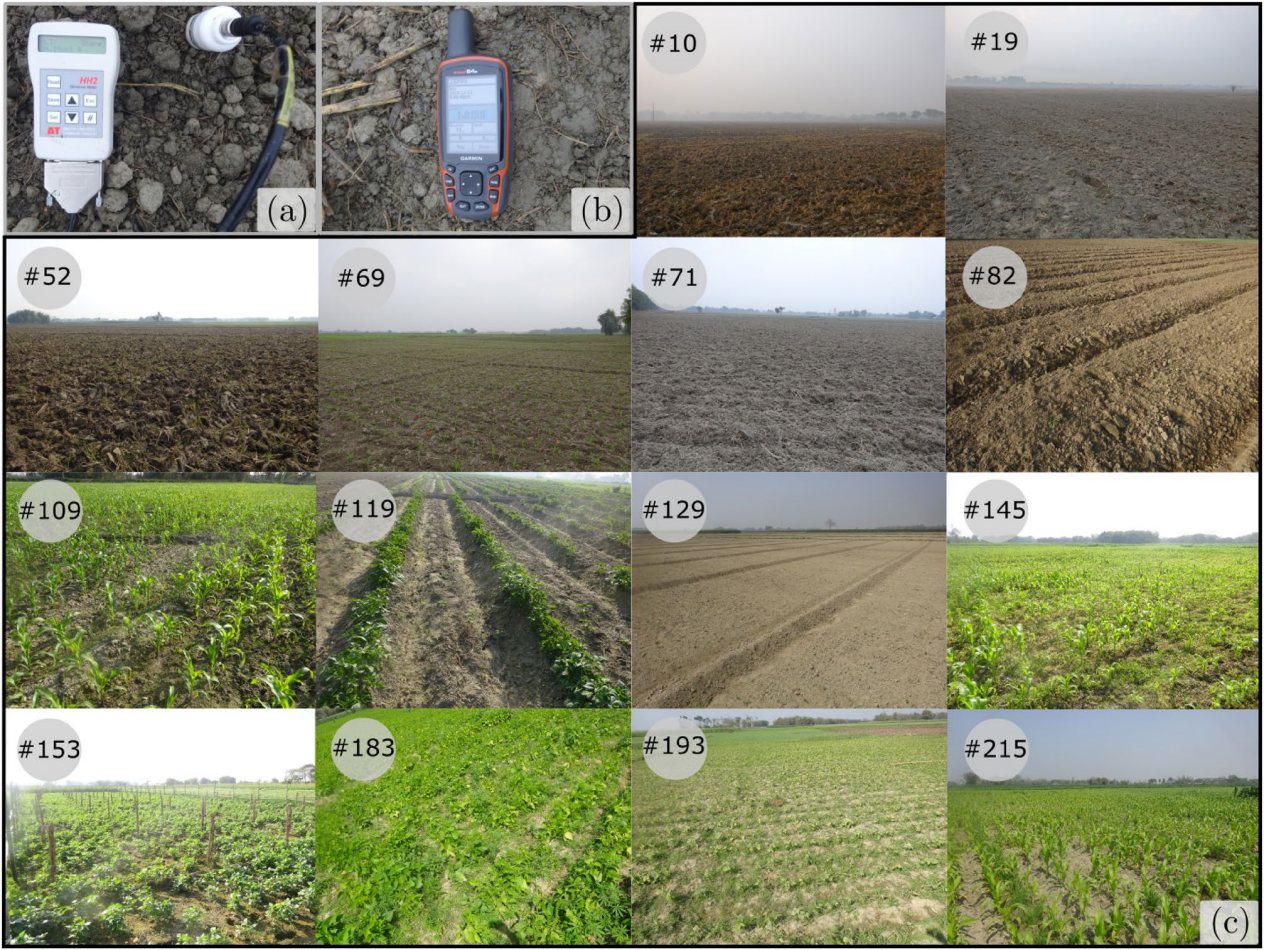
Field photographs illustrate the ground conditions (#10, #19, #52, #69, #71, #82, #109, #119, #129, #145, #153, #183, #193, #215) of the study area at the time of soil-moisture measurement .

The Sentinel-1A and 1B acquire images in strip map mode, interferometric wide swath mode, extra-wide swath mode, and wave mode. Depending upon the acquisition mode, the SAR products are available at three levels; level-0 (unfocused SAR raw data), level-1 (Single Look Complex (SLC) and Ground Range Detected (GRD) data), and level-2 (Ocean geophysical product derived from level-1). For wave mode, only single polarisation is available i.e., either VV or HH. For the remaining modes, dual polarised images are available i.e., either VV+VH or HH+HV. For the polar environment, sea-ice zone HH or HH+HV polarised data is available. The VV or VV+VH polarised data is available for all other observation zones at 10 m $\times$ 10 m cell size with 250 km swath. We have used VV+VH dual polarised GRD (level-1) images in this study.

In continuation of Sentinel missions, the ESA launched Sentinel-2A (on 23rd June 2015) and Sentinel-2B (on 7th March 2017) as a constellation of two polar-orbiting satellites. They provide data in two levels; level-1C (top-of-atmosphere corrected), and level-2A (bottom-of-atmosphere corrected)^69^. Sentinel-2 (A & B) together have a revisit time of 5 days^70^. They acquire images of the earth in 13 different spectral bands from Visible Near Infra-Red (VNIR) to Short Wave Infra-Red (SWIR) of the electromagnetic spectrum. For our purposes, we have used band-4 (Red; 665 nm) and band-8 (NIR; 865 nm). These bands have a spatial resolution of 10 m.

In a joint venture with National Geospatial-Intelligence Agency (DoD/NGA), the German Aerospace Center (DLR), and Agenzia Spaziale Italiana (ASI), NASA launched an 11 days SRTM shuttle mission in February 2000. It contained two independent SAR sensors in C-band (NASA) and X-band (DLR/ASI). Currently, three versions of SRTM are publicly available. This includes SRTM non-void filled, SRTM void filled, and SRTM 1 arc-second global^71^. We downloaded the void-filled DEM of spatial resolution 1 arc-second (30 m).

### Field measurement

In the field campaigns during December 11–20, 2019 and March 01–06, 2022, we measured soil moisture on the Kosi Fan by using the ML3 theta probe (Fig. 3a). We calibrated the theta probe by using the procedure explained in Singh et al.^14^. We adopted universal random grid sampling method to measure soil moisture in the field. We divided the study area into small square grids of 4 km $\times$ 4 km (Fig. 1) and randomly selected the grids for measurements. To ensure the same moisture content is illuminated and recorded by the Sentinel-1 SAR pulses, we measured the surface soil moisture at 5 cm depth from the topsoil layer^66–68^. To minimise the spatial heterogeneity, we collect 7–10 in-situ measurements over the footprint of the satellite pixel (i.e., 60 m). Each measurement is separated by at least 20 m. Finally, we calculate the average value of these measurements to get a representative value of soil moisture in a grid. This enables us to perform the direct point-to-pixel comparison of soil moisture^72,73^. We collected 224 such measurements over the entire study area from the apex to the toe of the Kosi Fan. At each sampling location, we have also recorded their coordinates using the Garmin GPSmap-64s (Fig. 3b). Figure 3c illustrates the ground conditions of some of the sampling sites (#10, #19, #52, #69, #71, #82, #109, #119, #129, #145, #153, #183, #193, and #215).

## Methodology

**Figure 4:**
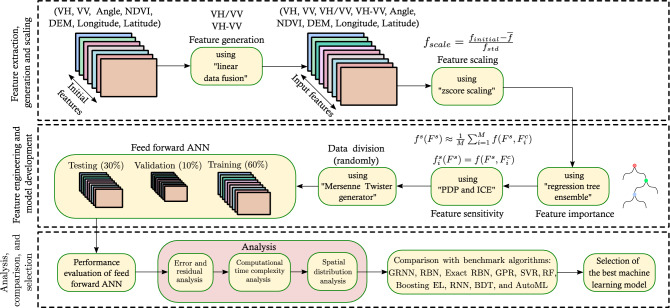
Flowchart illustrates the detailed methodology.

Figure 4 illustrates the detailed methodology adopted in this study. Firstly, we process the satellite images to extract input features to be used in the machine learning model. Secondly, we perform the feature engineering and setup a feed-forward multi-layer ANN model for training, validation, and testing. Finally, we evaluate the performance of ANN in terms of error analysis, computational time complexity analysis, and spatial distribution analysis and compare the output of the ANN model with ten different benchmark algorithms (i.e., GRNN, RBN, Exact RBN, GPR, SVR, RF, Boosting EL, RNN, BDT, and AutoML). The detailed feature extraction and model setup steps are discussed in the following subsections.

### Feature extraction

#### Image processing

We used the Sentinel Application Platform (SNAP v8.0) to process Sentinel-1 images. It is an open-source Earth Observation processing tool. We performed the radiometric calibration, multi-look correction (with a multi-looking factor of 6), speckle noise removal, and terrain correction to process the raw Sentinel-1 images. The resulting backscatter ($\sigma _0$) images for both polarisations (VV and VH) have the grid size of $\mathrm {60 \times 60 \; m}$. This is because of the multi-looking process, which averages the adjacent oblong pixels to a square pixel of size 60 m (raw pixel size multiplied by the multi-looking factor). We also processed the Sentinel-2 images to compute the normalised vegetation index (NDVI). We do this by taking a ratio of the difference between near-infra-red and red bands to their sum. The resulting NDVI image has a spatial resolution $\mathrm {10 \times 10 \; m}$. The NDVI image has pixel values between − 1 to + 1. The higher values of NDVI represent healthy vegetation^74^.

#### Feature selection and scaling

The prediction accuracy of any machine learning model primarily depends on the quality of input data. Without high-quality datasets, even high-performing machine learning algorithms are rendered ineffectual. In addition, the data pre-processing task is required to transform the raw data into a form that is more suitable to the machine learning model, which increases the efficiency and accuracy of the model. We have performed three feature engineering operations (i.e., feature extraction, generation, and scaling). Initially, we have extracted seven features from Sentinel-1, Sentinel-2 images, and DEM data. These are backscatter values ($\sigma _0$) in VV and VH polarisations, and incidence angles from Sentinel-1 images. The radar backscatters (VH and VV) are highly sensitive to soil moisture due to the presence of dielectric gradient^16^. The SAR incidence angle is an important sensor parameter that influences satellite-derived soil moisture^75,76^. We obtained the NDVI values from Sentinel-2 and elevation from the mean sea level at each pixel from the DEM. We also obtained the coordinates (Latitude and Longitude) of each pixel of the input images. Vegetation descriptors such as NDVI are important to incorporate the impact of vegetation on soil moisture retrieval. The dependency of soil moisture on the surface elevation is well known and frequently used in observing the soil moisture pattern and machine learning models^77–80^. To incorporate the spatial dependencies of data in spatial machine learning applications, we included geolocation (i.e., Latitude and Longitude) variables^50,81,82^. Further, we have also generated two synthetic features (i.e., VH/VV and VH-VV) from the existing feature set (i.e., VH and VV) by using the linear data fusion of VH and VV^39^. These synthetic features are more sensitive towards the dielectric and geometric properties of soil^76,83,84^.

We now use the nearest neighbour resampling method to resample the image pixels of the input features to a common grid size ($60 \times 60\;$ m). Finally, we apply the standard z-score scaling to scale all the nine features at the same level according to Eq. (1);

1 $$\begin{aligned} f_{scale} = \frac{f_{initial} - {\overline{f}} }{f_{std}} \end{aligned}$$

where $f_{initial}$ represent the initial feature, ${\overline{f}}$ represent the mean value, and $f_{std}$ is the standard deviation of the feature.

#### Feature importance and association

We evaluate the relevancy of our input features in predicting the response variable (i.e., soil moisture). We compute the importance score of individual features by using the regression ensemble tree approach. We boosted five hundred regression trees (i.e., *m* = 500), each with an unity learning rate (i.e., $\gamma$=1). We do this by using the Least Squares gradient Boosting (LSBoost) algorithm. We have considered the traditional decision tree as a weak learner (i.e., decision stumps). The LSBoost algorithm starts training a single weak learner at a time and simultaneously identifies its weak points. Based on these weak points, it creates a new weak learner ($h_{i}$) and computes the corresponding weight (i.e., $\delta _i$). Finally, the current model ($L_{i}$) is updated by the algorithm by focusing on the weak point of the previous weak learner ($L_{i-1}$) according to Eq. (2);

2 $$\begin{aligned} L_{i} = L_{i-1} + \gamma \cdot \delta _i \cdot h_{i} \quad \quad (i = 1, 2, 3, \ldots , m) \end{aligned}$$

After training, it incorporates the weak learner into the current model. It then iteratively generates the ensemble of weaker learners (i.e., a single strong learner, $L_m$). Now, we estimate the entire changes in node risk that result from splitting on each feature, normalising it in relation to the total number of branch nodes (i.e., $NR_{branch}$), and using that information, we compute the relative feature relevance score. Mathematically, the changes in the node risk (i.e., ${\Delta NR}$) is computed according to Eq. (3);

3 $$\begin{aligned} {\Delta NR} = \frac{NR_p - (NR_{c1}+NR_{c2})}{NR_{branch}} \end{aligned}$$

where $NR_p$ represents the node risk of the parent node and $NR_{c1}$ & $NR_{c2}$ represents the node risk of two children. The node risk at individual node ($NR_i$) is calculated according to Eq. (4);

4 $$\begin{aligned} NR_{i} = P_{i} \cdot MSE_{i} \end{aligned}$$

where $P_i$ and $MSE_i$ represents the node probability and mean square error of node *i*, respectively.

Further, to measure the correlation amongst the features, we calculate the feature association matrix (9 $\times$ 9 matrix). The existence of any correlated features adversely affects the machine learning model by making the model unstable and more sensitive to uncertainty. The values in the matrix represent the similarities between the decision rule that split on each observation. A higher value for a pair of features suggests that they are highly correlated.

#### Feature sensitivity

The feature importance score only suggests the relevancy of a feature with respect to the response variable (*i.e.,* soil moisture). To understand the association (either positive or negative) between the features and response variable, we need to analyse the Partial Dependency Plot (PDP), and Individual Conditional Expectation (ICE) curves^85,86^. PDP explains the partial dependency of each feature of the feature data (*i.e.,* $\varvec{F} = \{f_1, f_2, \ldots , f_n\}$, where *n* is the total number of features) on soil moisture by marginalising the impact of all other features. Whereas the ICE is an advancement of PDP that evaluates the feature impact on soil moisture for each observation. We created a subset $\varvec{F^s} = \{f_{1}\}$ and a complimentary set $\varvec{F^c}$ of $\varvec{F^s}$, any prediction on $\varvec{F}$ can be computed according to Eq. (5);

5 $$\begin{aligned} f(\varvec{F})=f \left( \varvec{F^s},\varvec{F^c}\right) \end{aligned}$$

We can now estimate the partial dependence of the feature in $F^s$ by computing the expectation ($E_c$) of Eq. (5);

6 $$\begin{aligned} f^s(\varvec{F^s})&= E_c \left[ f \left( \varvec{F^s},\varvec{F^c}\right) \right] \end{aligned}$$

7 $$\begin{aligned}&= \int f \left( \varvec{F^s},\varvec{F^c}\right) \cdot mp_c(\varvec{F^c})\cdot d\varvec{F^c} \end{aligned}$$

where $mp_c(\varvec{F^c})$ is the marginal probability of $\varvec{F^c}$ according to Eq. (8);

8 $$\begin{aligned} mp_c(\varvec{F^c}) \approx \int p \left( \varvec{F^s},\varvec{F^c}\right) \cdot d\varvec{F^s} \end{aligned}$$

Finally, we can compute the partial dependency of the feature in $\varvec{F^s}$ according to Eq. (9);

9 $$\begin{aligned} f^s \left( \varvec{F^s}\right) \approx \frac{1}{M} \sum _{i=1}^{M} f \left( \varvec{F^s},\varvec{F_i^c}\right) \end{aligned}$$

where *M* is the total observations. Finally, we disaggregate Eq. (9) to obtain the ICE curves according to Eq. (10).

10 $$\begin{aligned} f_i^s \left( \varvec{F^s}\right) = f \left( \varvec{F^s},\varvec{F_i^c}\right) \end{aligned}$$

### Model setup

#### Feed-forward ANN

In a feed-forward ANN model, the basic computations performed by each neuron are used to predict the model performance. This is a two-step process. In the first step, individual inputs of the neuron (i.e., input vector, $\varvec{x}$) and the corresponding weight values (i.e., weight vector, $\varvec{w}$) are combined together by a summation function. The output of a summation function is a dot product of weight vectors and input vectors (i.e., $\varvec{w\cdot x}$). A bias (or threshold) is added to the dot product forming the output (*f*) according to Eq. (11). In the second step, output (*f*) is fed into the argument of an activation (or transfer) function, which is then used to calculate a scalar value.

11 $$\begin{aligned} f=\sum {\varvec{w}\cdot \varvec{x}}+b \end{aligned}$$

**Figure 5:**
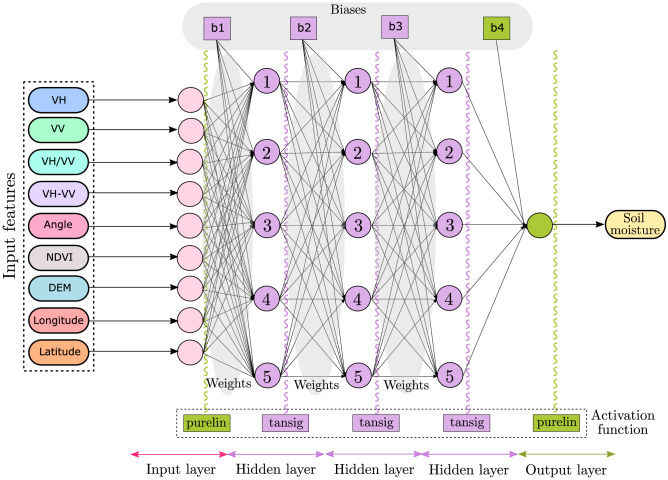
A fully connected 9-5-5-5-1 (I-$\mathrm {H_1}$-$\mathrm {H_2}$-$\mathrm {H_3}$-O) feed-forward ANN architecture for soil moisture estimation. I, H, and O represent the input, hidden, and output layers, respectively.

In multi-layer feed-forward ANNs, the network architecture consists of *N* neurons that are organised in *L* layers (*L* > 1). The first layer is input layer, which comprises input variables. Each neuron in a layer *l* (1 $\le$ *l* $\le$ L) is connected to all the neurons present in the previous layer ($l-1$) or to the input layer if; $l=1$ (Fig. 5). This way, the computation (or information) flows from the first to the last layer (forward propagation). The output layer ($l=L$) is the last layer of the neuron, whereas the other layers of the neuron are referred to as hidden layers. This study uses three hidden layers, each with five neurons. The neuron input is constructed as a linear combination of its received input values that correspond to the output of the previous layers^87^ according to Eq. (12);

12 $$\begin{aligned} {a^{l}=\sum \varvec{w}^{l}\cdot \varvec{x}^{l-1}} + b^{l} \end{aligned}$$

where, ${a^l}$ is the input of a neuron present at *l* layer, ${\varvec{w}^{l}}$ is the weight vector for the neurons present at *l* layer, ${\varvec{x}^{l-1}}$ is the output of a neuron present at $l-1$ layer, and ${b^{l}}$ is the bias value at layer *l* which is followed by an activation function.

#### Activation function

The choice of an activation function determines how a network maps the input features to the output^88,89^. Different layers can have different activation functions, and their selection can strongly influence the performance of a feed-forward ANN in terms of complexity and accuracy. However, there is no universal rule to select the activation functions. The identity (or linear) activation function (purelin) is almost always used at the input and output layers, whereas non-linear activation functions are generally preferred at the hidden layers. The most commonly used non-linear activation functions are hyperbolic tangent sigmoid (tansig) and log-sigmoid (log-sid)^90,91^.

We have used the identity activation function for the input and output layers and the hyperbolic tangent sigmoid activation function for all the hidden layers. Mathematically, the hyperbolic tangent sigmoid is expressed as Eq. (13);

13 $$\begin{aligned} T = \frac{2}{\left( 1+e^{-2\cdot n}\right) -1} \end{aligned}$$

This is mathematically analogous to tanh(n). We are using Eq. (13) instead of tanh(n) due to its lower time complexity. To reduce the computational time complexity, usually, the fast approximations of activation functions are used in deep learning^92^.

#### Training algorithm

Once the architecture of the feed-forward ANN model is ready, now we need to train the model. For getting the training data, we divided the complete data in a 60:10:30 ratio randomly using the Mersenne Twister generator for training, validation, and testing, respectively. During the training phase, we use the training algorithm to minimise the output error by updating the weights and biases. We have used the Levenberg-Marquardt (LM) backpropagation algorithm for training the feed-forward ANN model^93^. It is based on Newton’s method that was developed to optimise the sums of squares of the nonlinear functions. The Gauss-Newton approach itself has a limitation: the matrix might not be invertible. This problem can be resolved by modifying the Hessian matrix according to Eq. (14).

14 $$\begin{aligned} \varvec{G} = \varvec{H} + \mu \varvec{I} \end{aligned}$$

where ${\varvec{H}}$ represents the Hessian matrix, $\mu$ is a scalar that is the co-efficient of the steepest descent approach and Gauss-Newton method, and ${\varvec{I}}$ is the identity matrix. To minimise the complexity involved in the computation of the Hessian matrix, it is approximated by the Jacobian matrix, which is computationally less expensive according to Eq. (15). The LM updates the values of weights and biases through a Newton-like iterative approach given by Eq. (16).

15 $$\begin{aligned} \varvec{H}= & {} \varvec{J}^{T}\varvec{J} \end{aligned}$$

16 $$\begin{aligned} \varvec{w_{k+1}}= & {} \varvec{w_{k}} - \left[ \varvec{J}^{T}\varvec{J} + \mu \varvec{I}\right] ^{-1}\varvec{J}^{T}\varvec{e} \end{aligned}$$

where $\varvec{J}$ is the Jacobian matrix and $\varvec{e}$ is the network error vector.

**Figure 6:**
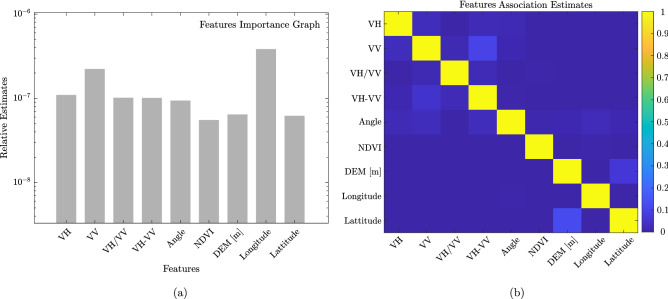
(**a**) The feature importance graph indicates the relative estimate of each feature obtained from the regression ensemble tree approach. The y-axis is in the log-scale. (**b**) Feature association graph indicating the correlation of each feature using heatmap.

**Figure 7:**
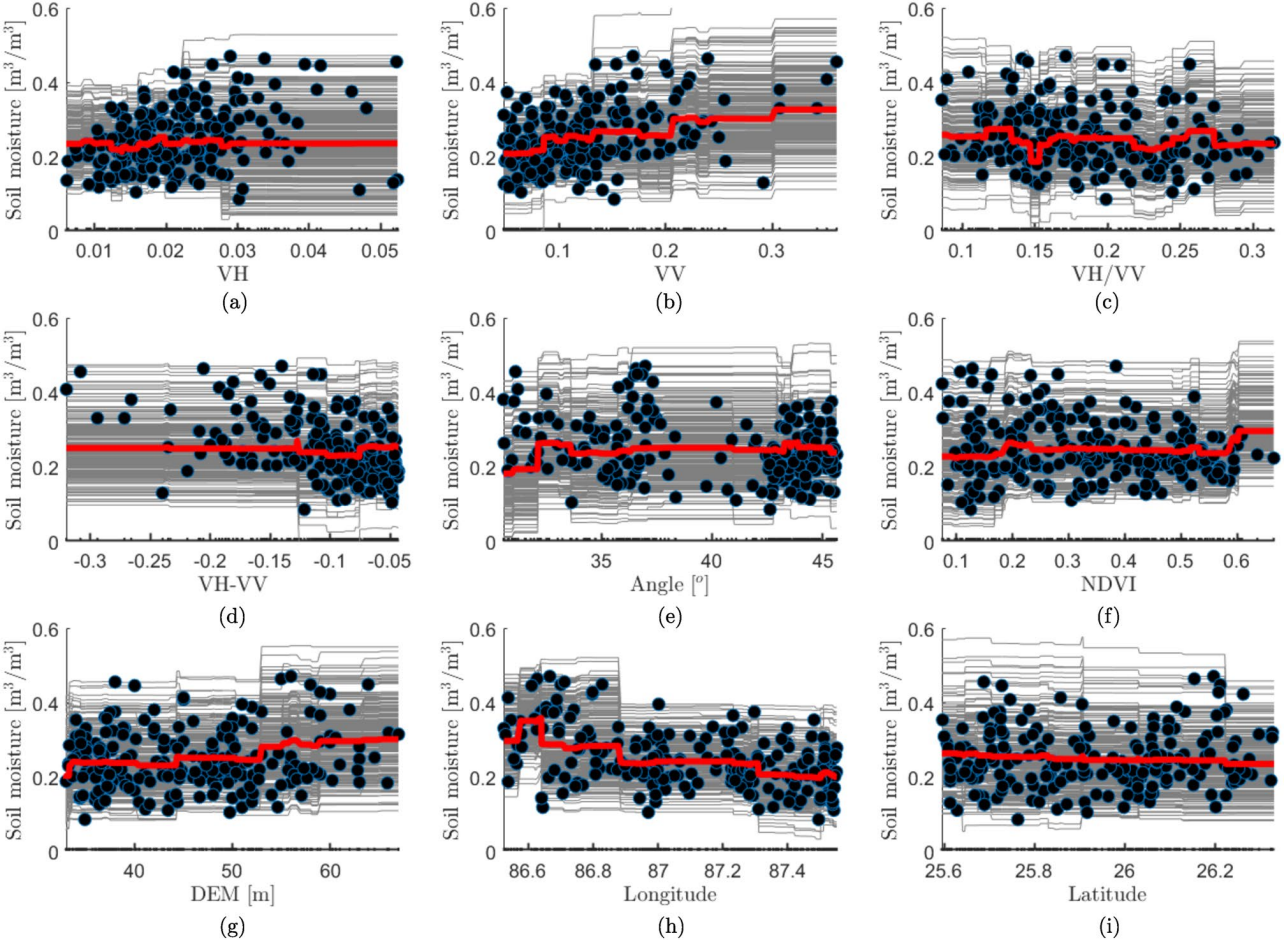
Feature sensitivity analysis using PDP (red line) and ICE curve (gray lines). The circles in black represent the observations.

## Results

### Feature importance and association

We plot the relative importance score of each feature (Fig. 6a). A high value of the feature importance indicates more predictive power (i.e., more relevant feature). We observed that Longitude, VV, and VH have a high feature importance score. A high contribution from the backscatter features (i.e., VV and VH) is in accordance with previous studies^94^. High contribution from Longitude indicates a possible control of the geolocation feature due to the morphology of the Kosi Fan. This point has been elaborated in the “Discussion” section. Interestingly, the synthetic features (i.e., VH/VV and VH-VV) that were generated through a linear data fusion have nearly the same importance score, which is relatively higher than the importance score of other input features such as incidence angle, NDVI, DEM, and Latitude. NDVI is relatively the least relevant feature with a minimum feature importance value.

Figure 6b is a heatmap of the feature association matrix. A high value in the feature association matrix indicates a high correlation among the features. We observed our features are not correlated. This indicates that the input features have appropriately trained the model without any instability and sensitivity.

### Feature sensitivity

To analyse the impact of individual input features on predicting soil moisture, we have constructed the Partial Dependency Plot (PDP) and Individual Conditional Expectation (ICE) curves (Fig. 7). We do not observe a clear positive trend in VH. This could be due to the presence of some high-value VH observations (> 0.04) corresponding to low soil moisture (< 0.2 $\mathrm {m^3/m^3}$). The presence of such limited oddity observations results in the dual behavior of VH, which is evident from the ICE curves. The majority of ICE curves corresponding to low-value VH (< 0.02) exhibit the same trend. However, we observed some ICE lines exhibit upward (after $\approx$ 0.025) while some (after $\approx$ 0.028) show downward trends. These trends get canceled out as the PDP takes the average of all the ICE lines, resulting a flat line after 0.03. The dual behavior in VH is generally observed, when soil moisture values are measured at the locations of high sub-pixel heterogeneity^95^. Such observations are inevitable while working with large and diverse in-situ measurements. The ICE curves of all other input features behave in a similar way without any significant deviation. This indicates that the PDP correctly illustrates the impact of all other features without concealing any local variations. We observed a strong positive impact of VV on soil moisture. VH/VV does not show any trend on soil moisture, whereas VH-VV has a slightly negative impact. The incidence angle and NDVI have undulated positive impact on soil moisture. An overall positive impact of DEM is observed. Longitude has a strong negative impact, whereas Latitude has a slight negative impact on soil moisture.

### Performance of the machine learning model

**Figure 8:**
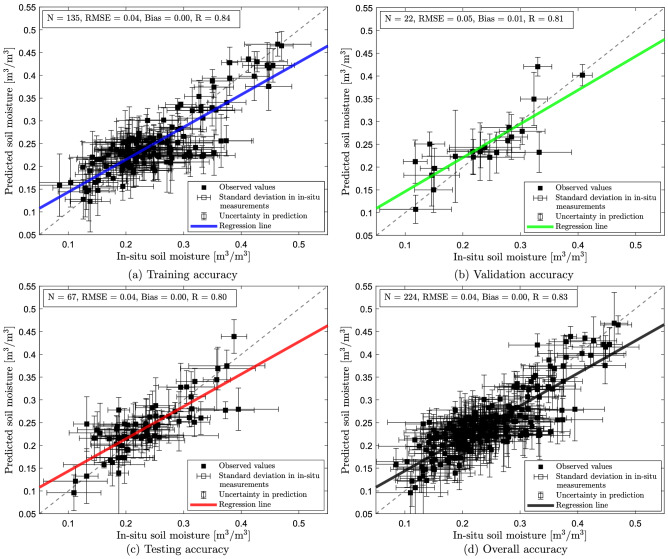
Model performance on (**a**) training, (**b**) validation, (**c**) testing, and (**d**) complete datasets. The dashed line in figure a-d represents y = x line. Horizontal error bar represents the standard deviation of the in-situ measurement, and vertical error bar represents the resulting uncertainty in the model prediction.

To estimate the goodness of the training process, we assess the performance of our trained feed-forward ANN model over the training data. On training data, we discovered that the model performed reasonably well with R = 0.84, bias = 0 $\mathrm {m^3/m^3}$, and RMSE = 0.04 $\mathrm {m^3/m^3}$ (Fig. 8a). However, assessing the model performance solely on the training data results in a bias observation. We need to evaluate the model performance by using unseen data (i.e., validation and testing data). We assess the model performance using the validation data while tuning the model parameters. We found a good agreement between the in-situ and predicted soil moisture for the validation process with R= 0.81, bias = 0.01 $\mathrm {m^3/m^3}$, and RMSE = 0.05 $\mathrm {m^3/m^3}$ (Fig. 8b). The presence of small positive bias indicates a slight overestimation of the trained model. We then fed the testing data into our model and evaluated the performance. We found the soil moisture measured in the field accorded well with the prediction with R = 0.80, bias = 0 $\mathrm {m^3/m^3}$, RMSE = 0.04 $\mathrm {m^3/m^3}$ (Fig. 8c). Finally, we report (Fig. 8d) the overall accuracy (R = 0.83, bias = 0 $\mathrm {m^3/m^3}$, and RMSE = 0.04 $\mathrm {m^3/m^3}$) of the trained model by evaluating its performance over the complete datasets (i.e., training+validation+testing).

#### Error and residual analysis

**Figure 9:**
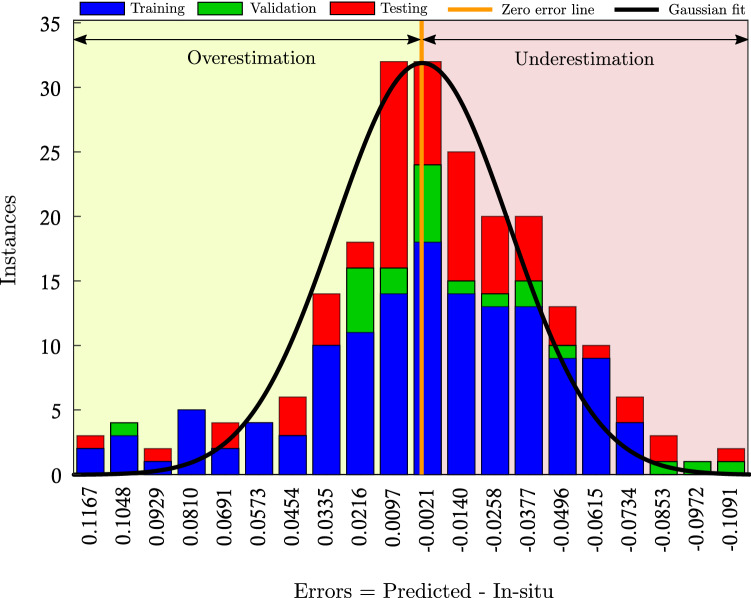
Error histogram (with 20 bin size) for training, validation, and testing phase. The area to the left and right of the zero error (orange) line represents overestimated and underestimated regions, respectively.

**Figure 10:**
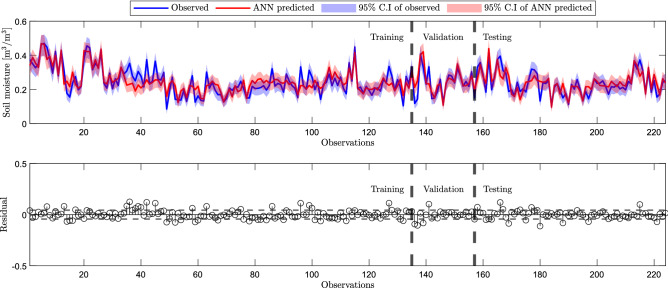
The top panel shows the line plot of observed and predicted soil moisture plotted for training, validation, and testing. The bottom panel shows the corresponding residual plot. The height of the vertical line with a circular cap represents the magnitude of the residual. The dashed line represents the overall RMSE value.

Figure 9 illustrates the error (i.e., predicted - in-situ) in the training, validation, and testing process. The height of the stacked bars represents the instances that occurred with the same error. We selected the bin size of twenty to represent the errors that range from − 0.1167 $\mathrm {m^3/m^3}$ (leftmost bin) to +0.1091 $\mathrm {m^3/m^3}$ (rightmost bin). The vertical orange line represents the zero error. The region left to this line represents the overestimation, and the region right to it is the underestimation. We have plotted a best fit Gaussian (curve in black) to the error histogram. Ideally, we expect the histogram to be normally distributed with zero mean. The distribution of our histogram is nearly normal, with the location of its peak at the zero error line indicating a good fit. Figure 10 shows the measured and predicted soil moisture at 95% confidence Interval (C.I). The predicted values of soil moisture accord well with the observed values. Further, to evaluate our model, we performed the residual analysis. We observed residuals are scattered randomly and do not show any pattern.

Finally, we performed the spatial distribution analysis. We formed thirty sets of training-validation-testing datasets by randomly dividing the in-situ observations (224) using Mersenne Twister random generator. We computed each set’s training, validation, and testing accuracy using the proposed network and reported the $\mu \pm \sigma$. We observed an overall steady response for the training (R: 0.80 $\pm 0.05$, RMSE: 0.05 ± 0.01 $\mathrm {m^3/m^3}$, and bias: 0.00 ± 0.00 $\mathrm {m^3/m^3}$), validation (R: 0.74 ± 0.08, RMSE: 0.05 ± 0.01 $\mathrm {m^3/m^3}$, and bias: 0.00 ± 0.01 $\mathrm {m^3/m^3}$), and testing accuracy (R: 0.72 ± 0.04, RMSE: 0.05 ± 0.01 $\mathrm {m^3/m^3}$, and bias: 0.00 ± 0.01 $\mathrm {m^3/m^3}$). This confirms the reliability and accuracy of the proposed network.

## Discussion

### Comparison with different scenarios of feed-forward ANN

We have generated different scenarios based on two themes for robust analysis. For this, we vary the number of hidden layers and the respective neurons in each hidden layer. We have generated twelve scenarios by varying the number of hidden layers from one to four, with single, five, or ten neurons in each layer (Table 2). We evaluate the performance of these scenarios in the training, validation, and testing phase with three performance metrics (i.e., R, RMSE, and bias). We evaluated an additional performance metric, namely RMSE-observations standard deviation ratio (RSR), which is widely used for performance rating (very good, good, satisfactory, and unsatisfactory category) in the field of hydrology^96^. RSR (Eq. 17) consists of an error index with a normalisation factor, it can be used in diverse constituents. The value of RSR ranges from zero (indicating a perfect model with zero RMSE) to a large positive value (indicating a poor model with high RMSE).

17 $$\begin{aligned} RSR = \frac{Overall_{RMSE}}{STD_{in-situ}} \end{aligned}$$

where $Overall_{RMSE}$ is the overall RMSE and $STD_{in-situ}$ is the standard deviation of the in-situ soil moisture measurements. We estimated the performance of the scenarios by different RSR categories; very good (0 $\le$ RSR $\le$ 0.50), good (0.50 < RSR $\le$ 0.60), satisfactory (0.60 < RSR $\le$ 0.70), and not satisfactory (RSR > 0.70). We observed that only the proposed scenario (i.e., 9-5-5-5-1) falls under the very good category. Out of twelve, two scenarios (i.e., 9-5-5-1 and 9-5-5-5-5-1) fall under the good category. Among these, we found that scenario 9-5-5-5-5-1 emerges as the best based on testing metrics. We have selected the best performing feed-forward ANN architecture (i.e., 9-5-5-5-1) to further generate ten different scenarios by varying the model inputs (Table 2). We observed that the performance of our architecture is optimal only when all nine features are considered.

**Table 2:** Comparison of different scenarios by varying input feature combination and network architecture (hidden layers with neurons).

Scenarios		Training			Validation			Testing			RSR	Interpretation
		**R**	**RMSE** $\mathrm {[m^3/m^3]}$	**Bias** $\mathrm {[m^3/m^3]}$	**R**	**RMSE** $\mathrm {[m^3/m^3]}$	**Bias** $\mathrm {[m^3/m^3]}$	**R**	**RMSE** $\mathrm {[m^3/m^3]}$	**Bias** $\mathrm {[m^3/m^3]}$		
Layers/Neurons variation	9-1-1	0.68	0.06	0.003	0.75	0.06	− 0.017	0.62	0.06	− 0.003	0.74	Not satisfactory
	9-5-1	0.83	0.04	0.002	0.65	0.05	0.010	0.70	0.06	0.008	0.64	Satisfactory
	9-10-1	0.79	0.05	0.002	0.66	0.06	0.001	0.60	0.06	0.007	0.68	Satisfactory
	9-1-1-1	0.65	0.05	0.000	0.87	0.05	-0.017	0.67	0.06	-0.018	0.70	Satisfactory
	9-5-5-1	0.90	0.04	0.001	0.82	0.05	-0.017	0.59	0.05	0.009	0.58	Good
	9-10-10-1	0.78	0.05	0.007	0.73	0.06	0.010	0.57	0.06	0.013	0.70	Satisfactory
	9-1-1-1-1	0.71	0.05	0.000	0.85	0.06	0.001	0.52	0.06	0.009	0.74	Not satisfactory
	9-5-5-5-1	0.84	0.04	0.001	0.81	0.05	0.011	0.80	0.04	0.004	0.50	Very good
	9-10-10-10-1	0.74	0.05	0.000	0.85	0.04	0.000	0.77	0.05	0.019	0.63	Satisfactory
	9-1-1-1-1-1	0.68	0.06	− 0.002	0.61	0.05	0.011	0.65	0.06	− 0.008	0.75	Not satisfactory
	9-5-5-5-5-1	0.81	0.04	0.007	0.88	0.04	0.003	0.73	0.06	0.007	0.60	Good
	9-10-10-10-10-1	0.86	0.04	− 0.005	0.56	0.05	− 0.014	0.53	0.06	-0.018	0.67	Satisfactory
Input features variation	VH	0.39	0.07	-0.001	0.28	0.08	− 0.014	0.33	0.07	-0.003	0.93	Not satisfactory
	VV	0.29	0.08	0.008	0.45	0.07	0.004	0.49	0.07	0.014	0.93	Not satisfactory
	VH, VV	0.46	0.07	0.018	0.45	0.08	0.008	0.03	0.08	0.019	0.94	Not satisfactory
	VH/VV	0.16	0.08	0.006	0.27	0.08	0.000	0.27	0.07	0.011	0.98	Not satisfactory
	VH-VV	0.48	0.07	0.001	0.41	0.07	− 0.000	0.62	0.06	− 0.001	0.87	Not satisfactory
	VH, VV, VH/VV, VH-VV	0.58	0.06	0.002	0.36	0.08	− 0.016	0.18	0.07	− 0.001	0.89	Not satisfactory
	VH, VV, VH/VV, VH-VV, angle	0.68	0.06	-0.009	0.38	0.07	− 0.004	0.46	0.08	0.002	0.82	Not satisfactory
	VH, VV, VH/VV, VH-VV, angle, NDVI	0.68	0.06	− 0.002	0.39	0.06	0.014	0.50	0.06	0.009	0.78	Not satisfactory
	VH, VV, VH/VV, VH-VV, angle, NDVI, DEM	0.82	0.04	0.001	0.64	0.07	-0.027	0.56	0.07	0.007	0.70	Satisfactory
	All nine features	0.84	0.04	0.001	0.81	0.05	0.011	0.80	0.04	0.004	0.50	Very good

### Comparison with the benchmark algorithms and AutoML approach

For a fair evaluation, we compared the performance of our fully connected feed-forward ANN with the other benchmark algorithms to predict soil moisture by using the same data. We used the GRNN, RBN, ERBN, GPR, SVR, RF, Boosting EL, RNN, and BDT as the potential benchmark algorithms^97–106^. Other than these benchmark algorithms, we have also compared our result with the recently emerged Automated Machine Learning (AutoML) model^107^. We fed the same datasets into the AutoML platform of MATLAB^®^ driven by *fitrauto* library. It automatically selects the machine learning model (i.e., linear regression, SVR, GPR, BDT, and EL) and optimise the corresponding tuning parameters through the Bayesian optimisation technique. During the optimisation process, it minimises the objective function ($\mathrm {log(1+CV_{MSE})}$; where $\mathrm {CV_{MSE}}$ is the cross-validation MSE) iteratively. We found that the proposed ANN architecture outperforms all the benchmark algorithms with R = 0.80, bias = 0.004 $\mathrm {m^3/m^3}$, and RMSE = 0.040 $\mathrm {m^3/m^3}$ (Table 3).

To perform a robust and accurate comparison, we performed a statistical significance analysis to measure the performance of different ML models. To do so, we calculated the error in the predicted soil moisture (i.e., predicted - in-situ) for all the ML models. We applied statistical tests (i.e., Kolmogorov Smirnov and Shapiro-Wilk/Francia) to check the normality of the errors for each ML model. We found that the errors in each model are normally distributed. We then performed one-way ANOVA (ANalysis Of VAriance) to test a null hypothesis (i.e., $\mathrm {h_o}$: mean of the error distribution of all ML models are equal). Based on the result of the ANOVA test, we rejected the null hypothesis. We noticed that two ML models (i.e., ERBN and Boosting EL) have significantly different mean values from the feed-forward ANN. In addition, we found that the RNN and feed-forward ANN have nearly the same mean (i.e., not significantly different), indicating similar performance. This is in accordance with the interpretation we drew from the analysis of performance metrics (i.e., bias).

**Table 3:** Comparison with the benchmark algorithms (GRNN, RBN, Exact RBN, GPR, SVR, RF, Boosting EL, RNN, and BDT ) and AutoML approach.

Performance metrics	Machine learning models										
	Feed forward ANN	GRNN	RBN	ERBN	GPR	SVR	RF	Boosting EL (LSBoost)	RNN	BDT	AutoML
**R**	0.80	0.24	0.18	0.12	0.28	0.13	0.19	0.16	0.24	0.19	0.37
RMSE [$\mathrm {m^3/m^3}$]	0.040	0.060	0.061	0.062	0.060	0.062	0.061	0.061	0.059	0.048	0.048
Bias [$\mathrm {m^3/m^3}$]	0.004	− 0.014	− 0.006	− 0.048	− 0.026	− 0.001	− 0.011	− 0.037	0.004	0.024	− 0.023

**Figure 11:**
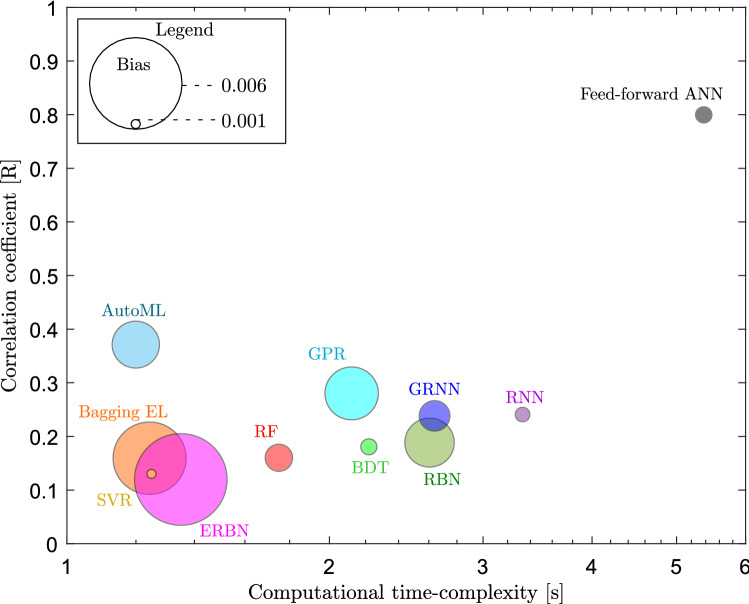
Comparison of the computational time complexity and performance of the benchmark algorithms (each represented in a different colour). The radius of the circle represents the magnitude of the bias for each model.

We have evaluated the computational time complexity (using CPU 64-GB, @3.3 GHz, 10-cores) of the feed-forward ANN model and compared it with the benchmark algorithms (Fig. 11). The computational time complexity of a fully connected feed-forward ANN is ${\mathcal {O}} \left( \alpha {n_l}_1 + {n_l}_1 {n_l}_2 + \cdots \right)$, where $\alpha$ is the number of features and ${n_l}_i$ is the number of neurons present at layer *i*^108^. We plotted the average computation time taken by each algorithm. We observed a clear trade-off between model performance and computational time complexity among these algorithms. The computational time complexity of the feed-forward ANN is slightly higher than the other algorithms. This is probably due to the large number of computations involved during the optimisation of the hyperparameters (i.e., weights and biases) by using the LM backpropagation algorithm.

**Figure 12:**
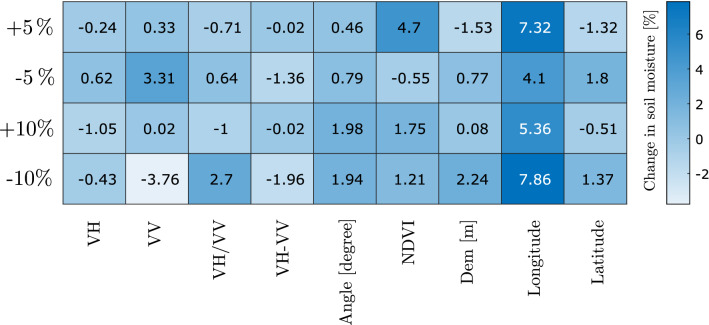
Sensitivity of feed-forward ANN architecture (9-5-5-5-1) by considering uncertainties (∓ 5% and ∓ 10%) in input features.

### Sensitivity analysis

Sensitivity analysis is important to assess the consistency of a data-driven model. We evaluated the response of our feed-forward ANN model concerning the uncertainty in the input features. In doing so, we introduce ±5%, and ±10% uncertainty in all the input features at a time by keeping other features unchanged and evaluating how these uncertainties from individual features contribute to the total uncertainty in the response variable (i.e., soil moisture). We observed for ±5%, and ±10% uncertainty in the input features, the uncertainty in the model-derived soil moisture ranges between $\approx$ − 4% and $\approx$ + 8% (Fig. 12). The model is more resistant to the presence of uncertainty in VH, VH-VV, incidence angle, and Latitude. In contrast, it is less resistant to the presence of uncertainty in NDVI and Longitude.

### Soil moisture on the Kosi Fan

**Figure 13:**
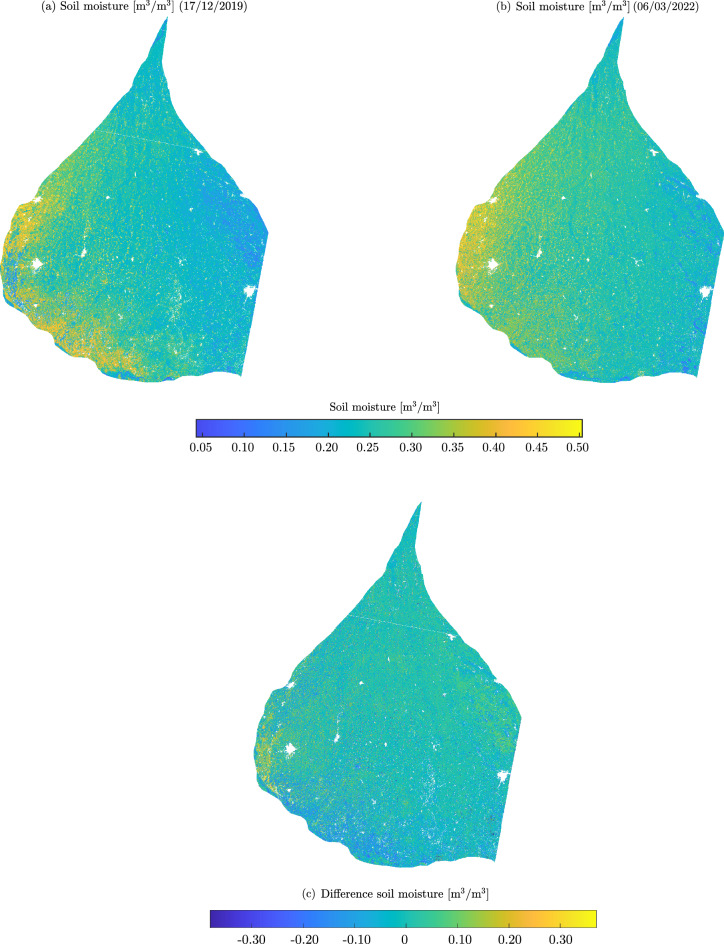
(**a**) High spatial resolution (i.e., 60 m) surface soil moisture map for $\mathrm {11^{th}}$ December 2019 and (**b**) $\mathrm {06^{th}}$ March 2022 (**c**) Corresponding difference soil moisture map. The transparent pixels represent the invalid regions.

Figures 13a and b are the surface soil moisture maps generated from the ANN for two different time frames (i.e., 17 December 2019 and 06 March 2022). The invalid regions (dense built-up and water bodies) have been masked.

**Figure 14:**
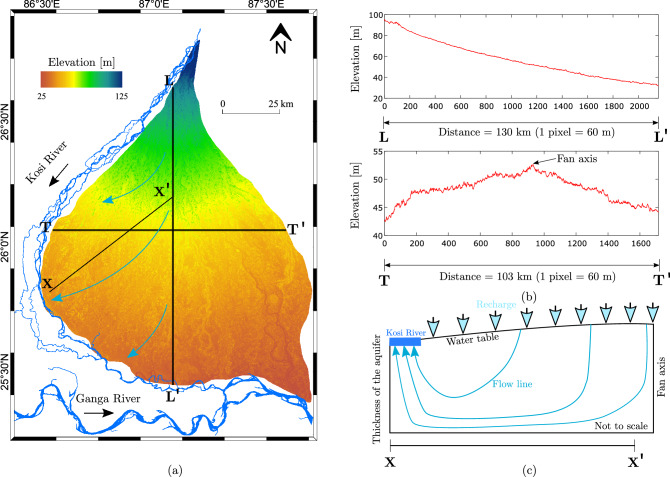
(**a**) Digital elevation model of the Kosi Fan, (**b**) elevation profiles on the fan along the longitudinal (LL') and transverse (TT') transects, and (**c**) schematic of the groundwater flow lines from the fan axis towards the western margin of the fan (modified after Khan et al.^109^).

On the Kosi Fan, soil moisture appears relatively high at the western margin. It is important to note that the Kosi River flows at the western margin. To understand the spatial variation of soil moisture, we performed a topographic analysis. A transverse (TT’) transect drown on a DEM exhibits a convex-upward profile of the Kosi Fan (Fig. 14). The elevation is maximum at the fan axis and decreases towards the western and eastern margins of the fan. This inherent topography of fan controls the drainage organisation. The drainage networks diverge from the fan axis towards the fan margins. Also, the groundwater table appears to follow surface topography. The water table is at a shallow depth in close proximity to the Kosi River (Fig. 14c). The topography, drainage orientation, and shallow water table at the western margin of the fan make this region prone to high soil moisture content.

Further, to assess the spatio-temporal variation of soil moisture content, we take the difference of soil moisture maps of two different dates (Fig. 13c). We observed high variability in the moisture content. This suggests a strong seasonal variability of soil moisture content on the fan surface. Processing long-time series microwave images would help to quantify the inter-annual variation and also to understand the impact of climate change and human perturbation on the spatio-temporal dynamics of soil moisture. Such analysis would be very useful to predict soil moisture conditions in the near future, which would help to plan agriculture and food security in the region.

## Conclusion

We applied a fully connected feed-forward (i.e., 9-5-5-5-1) ANN algorithm and data fusion to estimate surface soil moisture on the Kosi alluvial fan using the multi-sensor remote sensing images. From the input features, the Longitude, VV, and VH have emerged as the most relevant features for mapping surface soil moisture. Among these, Longitude and VV exhibit negative and positive impacts on soil moisture, respectively. We do not observe a clear positive trend for VH. Since the in-situ measurement of larger sample size usually contains few oddity samples, primarily from a location of large sub-pixel heterogeneity and dense vegetation. These samples usually return high VH values for low soil moisture resulting in a dual behaviour of VH^95^. We noticed DEM has a clear positive impact on soil moisture, whereas Latitude has a negative impact on soil moisture. The incidence angle and NDVI have a fluctuating positive impact, and VH-VV has a fluctuating negative impact on soil moisture. VH/VV did not show any clear trend. The spatial pattern of the surface soil moisture over Kosi Fan indicates a possible control of surface topology and fan morphology.

ANN with three hidden layers having five neurons (i.e., 9-5-5-5-1) each has a relatively high predictability of surface soil moisture than the other benchmark algorithms. However, there is a trade-off between the performance and computational time complexity. The fully connected feed-forward ANN has the highest time complexity with the best performance. This model is relatively more sensitive towards the presence of small uncertainly in the graphical indicator and geolocation features (i.e., NDVI and Longitude) than other input features.

This comprehensive framework allows us to generate a surface soil moisture map from dual polarised backscatter images from Sentinel-1, red and near-infrared surface reflectance from Sentinel-2, and DEM from SRTM satellite images. The outcome of this study could be used as input data to study waterlogging, flood inundation, agronomy, drainage congestion, drought prediction, and other hydrological applications.

## Data Availability

The computer algorithms originated during the current study can be made available from the corresponding author on a reasonable request.

## Data availibility

The datasets used and/or analysed during the current study available from the corresponding author on reasonable request.
